# Reversal of endocrine resistance in breast cancer: interrelationships among 14-3-3ζ, FOXM1, and a gene signature associated with mitosis

**DOI:** 10.1186/bcr2913

**Published:** 2011-06-29

**Authors:** Anna Bergamaschi, Barbara L Christensen, Benita S Katzenellenbogen

**Affiliations:** 1 Department of Molecular and Integrative Physiology, University of Illinois and College of Medicine at Urbana-Champaign, 524 Burrill Hall, 407 South Goodwin Avenue, Urbana, IL, 61801, USA

**Keywords:** estrogen receptor, antiestrogens, endocrine resistance, gene expression, 14-3-3ζ

## Abstract

**Introduction:**

Despite the benefits of estrogen receptor (ER)-targeted endocrine therapies in breast cancer, many tumors develop resistance. 14-3-3 ζ/YWHAZ, a member of the 14-3-3 family of conserved proteins, is over-expressed in several types of cancer, and our previous work showed that high expression of 14-3-3ζ in ER-positive breast cancers was associated with a poor clinical outcome for women on tamoxifen. Therefore, we now probe the role of 14-3-3ζ in endocrine resistance, and we examine the functional dimensions and molecular basis that underlie 14-3-3ζ activities.

**Methods:**

From analyses of four independent breast cancer microarray datasets from nearly 400 women, we characterized a gene signature that correlated strongly with high expression of 14-3-3ζ in breast tumors and examined its association with breast cancer molecular subtypes and clinical-pathological features. We investigated the effects of altering 14-3-3ζ levels in ER-positive, endocrine sensitive and resistant breast cancer cells on the regulation of 14-3-3ζ signature genes, and on cellular signaling pathways and cell phenotypic properties.

**Results:**

The gene signature associated with high 14-3-3ζ levels in breast tumors encompassed many with functions in mitosis and cytokinesis, including aurora kinase-B, polo-like kinase-1, CDC25B, and BIRC5/survivin. The gene signature correlated with early recurrence and risk of metastasis, and was found predominantly in luminal B breast cancers, the more aggressive ER-positive molecular subtype. The expression of the signature genes was significantly decreased or increased upon reduction or overexpression of 14-3-3ζ in ER-positive breast cancer cells, indicating their coregulation. 14-3-3ζ also played a critical role in the regulation of FOXM1, with 14-3-3ζ acting upstream of FOXM1 to regulate cell division-signature genes. Depletion of 14-3-3ζ markedly increased apoptosis, reduced proliferation and receptor tyrosine kinase (HER2 and EGFR) signaling, and, importantly, reversed endocrine resistance.

**Conclusions:**

This study reveals that 14-3-3ζ is a key predictive marker for risk of failure on endocrine therapy and serves a pivotal role impacting growth factor signaling, and promoting cell survival and resistance to endocrine therapies. Targeting 14-3-3ζ and its coregulated proteins, such as FOXM1, should prove valuable in restoring endocrine sensitivity and reducing risk of breast cancer recurrence.

## Introduction

Approximately 70% of breast cancers are positive for estrogen receptor (ER) α at diagnosis, and these patients often benefit from endocrine therapies that target ER, because the proliferative drive of these tumors and many of their phenotypic properties result from estrogens acting through the ER [1]. ER is a master regulator of gene expression in breast cancer, upregulating survival and proliferation-promoting factors and downregulating proapoptotic and tumor suppressing factors [1-4]. Endocrine therapies in breast cancer, when effective, are desirable because they are generally well tolerated and avoid the morbidity associated with radiation and chemotherapies.

All forms of endocrine therapies, including ER antagonists such as the selective estrogen receptor modulators (SERMs) tamoxifen and raloxifene, and the selective ER downregulator (SERD) fulvestrant, function by interrupting estrogen signaling through the ER. These therapies targeting the ER have profoundly impacted breast cancer treatment and improved patient survival [5]. The benefit of endocrine therapies, however, is limited by the development of resistance, a process that appears to result from upregulation of growth factor and protein kinase signaling pathways that provide an alternate mechanism in support of tumor cell proliferation and survival [6-9]. Hence, there is great interest in identifying and targeting, by inhibition or downregulation, key factors that mediate endocrine resistance.

We previously identified 14-3-3ζ, also known as YWHAZ, from gene expression profiling on a cohort of ER-positive breast tumor samples and found that women whose tumors had high levels of 14-3-3ζ showed a poor clinical outcome on tamoxifen [10]. However, the molecular mechanisms underlying this poor clinical response on endocrine therapy still remain unknown. 14-3-3ζ is a member of a highly conserved family of 14-3-3 proteins, and it functions as a scaffold or platform that regulates the activity and stability of interacting proteins by binding to their phosphoserine and phosphothreonine motifs [11-15]. Therefore, we have undertaken studies to probe the functional dimensions of 14-3-3ζ activity and its mechanistic basis in which we: characterize a gene signature associated with overexpression of 14-3-3ζ in breast tumors; determine the association of 14-3-3ζ with the molecular subtypes and clinical pathological features of breast cancers; identify the gene regulations, cellular pathways, and cell phenotypic properties modulated by 14-3-3ζ status; and examine the role of 14-3-3ζ in breast cancer endocrine resistance. Our studies reveal that 14-3-3ζ is a key survival factor integrating proliferative inputs from multiple cellular pathways, and that downregulation of 14-3-3ζ can restore endocrine sensitivity in resistant breast cancer cells. The findings suggest that targeting 14-3-3ζ or the proteins it regulates could be a useful approach for enhancing and prolonging the effectiveness of endocrine therapies.

## Materials and methods

### Analysis of microarray datasets and identification of a 14-3-3ζ gene signature

Microarray gene expression analysis and data processing were from four independent clinical studies encompassing 390 ER-positive primary breast tumors [10,16-18]. From the Frasor *et al*. dataset [10], we included the 67 ER-positive tumors from patients who subsequently underwent endocrine therapy with tamoxifen and microarrays were analyzed as described therein. From the van't Veer et al. dataset, we included 47 ER-positive breast tumors and associated expression data, and clinical data were obtained from Rosetta Inpharmatics (Kirkland, WA, USA) [17]. Downloaded log base 2 data were transformed to linear values and uploaded to GeneSpring GX 7.3 (Agilent Technologies, Santa Clara, CA, USA) From the Wang *et al*. dataset [18], we included 209 ER-positive breast tumors, and gene expression and clinical data were obtained from GEO (Series GSE2034). The downloaded data were transformed into GeneSpring GX 7.3 and chips and genes were median normalized and median polished. Log base 2 data from 67 ER-positive primary breast tumors from the Sorlie *et al*. cohort [16] were downloaded from GEO (Series 4335), uploaded to GeneSpring GX 7.3 and then chips and genes were median normalized. Frasor *et al*. [10] and Wang *et al*. [18] used the Hu133A-Affymetrix microarray platform; van't Veer *et al*. [17] used Hu25K-Agilent arrays; and Sorlie *et al*. [16] used cDNA Stanford arrays containing 8,102 genes. For the Sorlie *et al*. dataset, all the patients were treated with either doxorubicin or 5-fluorouracil and mitomycin C but no information on hormonal or other neo-adjuvant treatment was available. For Wang *et al*. and van't Veer *et al*., no treatments were publicly available or could be associated with any samples.

Hierarchical clustering of data was performed and displayed using Eisen Cluster and TreeView software for analysis and visualization. Based on 14-3-3ζ microarray expression levels, breast cancer samples [10] were divided into high (≥1.8 log2) and low (< 1.8 log2) 14-3-3ζ expression groups and a two-class statistical analysis of microarrays (SAM) was conducted [19]. Genes with FDR (false discovery rate) of 0.01 or less and with a fold change of three or more were included in the gene signature. The prediction analysis of microarrays method [20] was used as a cross-validation of the 14-3-3ζ signature.

### Survival analysis

Patients were divided into high and low 14-3-3ζ gene signature expression groups and Kaplan-Meier curves were computed by the Cox-Mantel log-rank test in WinStat for Microsoft Excel R. Fitch, Germany).

### Cell cultures and generation of stable cell lines

MCF7 cells, from the American Type Culture Collection (Manassas, VA, USA), and tamoxifen-resistant MCF-7 cells [21] were grown and treated as described [2,10]. Cells with stable knockdown of 14-3-3ζ (KD cells) were generated by transfection of pRNATin 5.1 (Ambion Austin, TX, USA) containing shRNA (TCTTGAGGTGGCCAATATTTC) targeting the 3' UTR. Cells were selected in the presence of hygromycin B (100 μg/ml). Some transfections utilized an adenovirus-mediated method [22].

### Western blot analysis

Whole-cell extracts were prepared using 1X RIPA Lysis buffer (Upstate/Chemicon Billerica, MA, USA) supplemented with 1X complete protease inhibitor (Roche, Basel, Switzerland). Western blotting used antibodies against 14-3-3ζ (Santa Cruz Biotechnology, Santa Cruz, CA, USA), β-actin (Sigma-Aldrich, St Louis, MO, USA), phosphoepidermal growth factor receptor (EGFR), phospho- human epidermal growth factor receptor 2 (HER2), phospho- mitogen activated protein kinase (MAPK) and phospho-AKT/PKB (protein Kinase B) (Cell Signaling, Danvers, MA, USA).

### RT-PCR and quantitative PCR

Total RNA was isolated from cells using TRIzol, reverse transcribed by SuperScript II reverse transcriptase (Invitrogen, Carlsbad, CA, USA), and real-time PCR performed on the ABI Prism 7900HT using SYBR Green PCR Master Mix (Applied Biosystems, Carlsbad, CA, USA) [10,23].

### Cell proliferation, colony formation and apoptosis assays

The WST-1 assay was used to quantify cell viability (Roche, Basel, Switzerland) and absorbance was measured at 450 nm using a BioRad 680 Microplate Reader (BioRad, Hercules, CA, USA). All assays were performed in triplicate. For the colony formation assay, a 1.5 mL base layer of agar (0.5% agar in phenol red-free DMEM with 5% charcoal stripped-fetal calf serum) was allowed to solidify in a six-well flat-bottomed plate before the addition of 1.5 mL of cell suspensions containing 4,000 cells in 0.35% agar in phenol red-free DMEM with 5% charcoal stripped-FCS. The cell-containing layer was then solidified at 4°C for 20 minutes. Colonies were allowed to grow for 15 days at 37°C with 5% CO2 before imaging and counting. Apoptosis was monitored based on DNA content by flow cytometry using BD-FACS Canto. Cells were fixed in 70% ethanol, stained for 30 minutes with 20 ug/ml propidium iodide (PI, Molecular Probe, Carlsbad, CA, USA) in Triton-X (Sigma, St Louis, MO, USA) in presence of DNAse-free RNAse A, and PI staining was measured [24].

## Results

### A gene signature and molecular phenotype in primary breast tumors associated with overexpression of 14-3-3ζ

**Figure 1:**
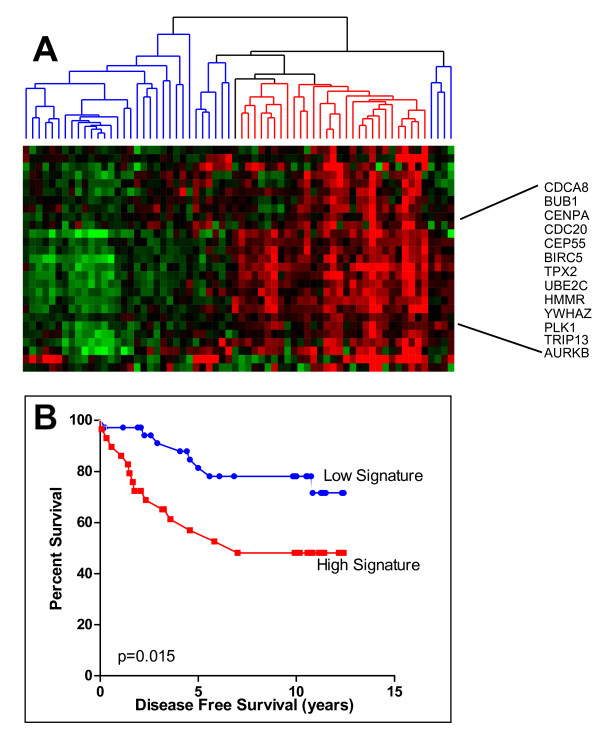
**Identification of a 14-3-3ζ gene signature**. **(a) **29 genes associated with high levels of 14-3-3ζ were identified and clustered based on their expression profile. Hierarchical clustering identified a subgroup of patients (red) characterized by elevated levels of mitosis and cytokinesis related genes. **(b) **Kaplan-Meier curves for the red and blue clusters of the hierarchical diagram in panel (a) distinguished between good (blue, signature low expression) and poor prognosis (red, signature high expression) patients (*P *= 0.015).

We previously reported that *trans*-hydroxytamoxifen specifically regulated the expression of a set of approximately 70 genes in ER-positive breast cancer cells. Of these, high 14-3-3ζ was associated with a poor clinical outcome for women on tamoxifen therapy [10]. To elucidate the role that 14-3-3ζ plays in engendering this poor clinical outcome, we sought to identify genes significantly associated with high level expression of 14-3-3ζ and to relate these to breast cancer phenotype and gain mechanistic insights into the functions of 14-3-3ζ. For this, we classified samples from our previously described cohort of 67 ER-positive primary breast tumors from women treated with tamoxifen [10] into two groups based on high or low 14-3-3ζ expression and employed two-class SAM analysis and retrieved 29 genes with an FDR of 0.01 or less and a fold change of three or more (Table 1). Using the DAVID database [25] to classify our signature gene list based on Gene Ontology terms, we found that 46% of the genes in this signature were significantly enriched in the "cell cycle" category (*P *≤ 0.0001). Among these were BUB1 (budding uninhibited by benzimidazoles 1 homolog), BIRC5/Survivin, CDCA8 (cell division cycle-associated protein 8), AURKB (aurora kinase B), CDC25B (cell division cycle 25 homolog B), and PLK1 (polo-like kinase 1), genes involved in mitosis and cytokinesis that tightly clustered with 14-3-3ζ (Figure 1a).

**Table 1:** List of genes in the 14-3-3ζ gene signature, based on SAM analysis

Symbol	Name	UGRepAccession
AURKB	Aurora kinase B	CD049340
BIRC5	Effector cell peptidase receptor 1	NM_001012271
BUB1	BUB1 budding uninhibited by benzimidazoles 1 homolog	AF053305
CDC20	Cell division cycle 20 homolog	BG256659
CDC25B	Cell division cycle 25 homolog B	NM_021873
CDCA8	Cell division cycle associated 8	BC000703
CENPA	Centromere protein A	BM911202
CEP55	Centrosomal protein 55kDa	NM_018131
CKS2	CDC28 protein kinase regulatory subunit 2	BQ898943
CYC1	Cytochrome c-1	BF569085
DGAT1	Diacylglycerol O-acyltransferase homolog 1	XM_001719374
EXOSC4	Exosome component 4	BM911415
FAM82B	Family with sequence similarity 82, member B	NM_016033
GPR172A	G protein-coupled receptor 172A	CR625605
HMMR	Hyaluronan-mediated motility receptor (RHAMM)	AF032862
HSPB8	Heat shock 22kDa protein 8	NM_014365
KPNA2	Karyopherin alpha 2 (RAG cohort 1, importin alpha 1)	BC067848
NDRG1	N-myc downstream regulated gene 1	NM_006096.3
PCSK1N	Proprotein convertase subtilisin/kexin type 1 inhibitor	BM805628
PLK1	Polo-like kinase 1	AB209179
RECQL4	RecQ protein-like 4	BC020496
SLC16A3	Solute carrier family 16, member 3 (monocarboxylic acid transporter 4)	NM_001042422
SLC39A4	Solute carrier family 39 (zinc transporter), member 4	AK056900
SQLE	Squalene epoxidase	NM_003129
TPX2	TPX2, microtubule-associated, homolog	NM_012112
TRIP13	Thyroid hormone receptor interactor 13	NM_004237
UBE2C	Ubiquitin-conjugating enzyme E2C	BC032677
UBE2S	Ubiquitin-conjugating enzyme E2S	BM479313
YWHAZ	Tyrosine 3-monooxygenase/tryptophan 5-monooxygenase activation protein, zeta polypeptide	BC051814

To analyze further how these genes might help explain the molecular phenotype of tumors overexpressing 14-3-3ζ, we performed unsupervised hierarchical clustering analysis and identified two main groups of patients based on 14-3-3ζ signature gene expression. When Kaplan-Meier analysis was performed using relapse as an endpoint, patients with breast tumors having high expression of these genes (High Signature Expression) showed a significantly poorer outcome (Figure 1b).

**Figure 2:**
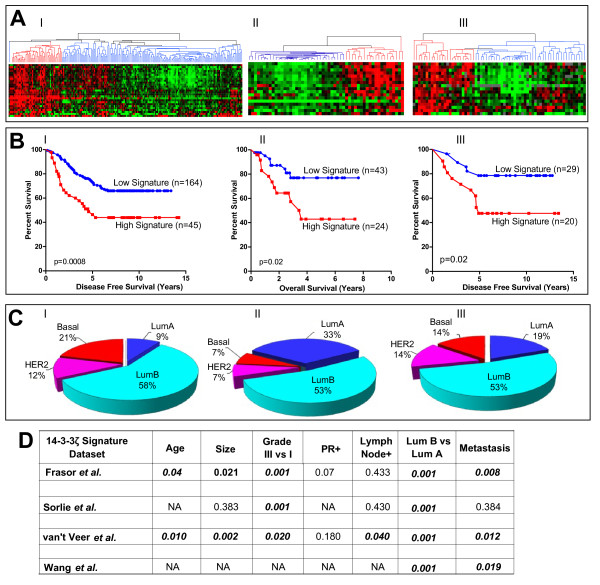
**Association of signature gene expression with prognosis, breast cancer molecular subtypes, and clinical-pathological features**. **(a) **Heat maps and dendrograms for Wang *et al*. (panel I), van't Veer *et al*. (panel II), and Sorlie *et al*. (panel III) datasets and expression of the 14-3-3ζ signature genes. From left to right, I) analysis of Wang *et al*. dataset comprising 209 estrogen receptor (ER)-positive breast tumors, II) analysis of 49 ER-positive tumors from the van't Veer *et al*. study and III) analysis of 67 ER-positive tumors from the Sorlie *et al*. study. **(b) **Kaplan-Meier survival curves of patient groups from three independent datasets of ER-positive breast tumors [16-18] based on expression patterns of the 14-3-3ζ gene signature. **(c) **Subtype classification of tumors with high expression of the signature genes, based on the five breast cancer molecular subtypes. Data were derived from three independent datasets of ER-positive breast tumors in panel a [16-18], as described in Materials and Methods. **(d) **Association of the 14-3-3ζ gene signature (*n *= 29 genes) with breast cancer clinical-pathological features. Pearson correlation values are shown. Numbers in bold indicate significant correlations, *P *< 0.05. NA, not available.

To further assess the relevance and applicability of this 14-3-3ζ signature, we selected ER-positive tumors from three other independent breast tumor microarray datasets with clinical information available. For all these three datasets no information on hormonal treatment was reported. Figure 2a shows the heat maps and dendrograms for expression of the 14-3-3ζ signature genes from these three studies (panel I Wang *et al*., panel II van't Veer *et al*., panel III Sorlie *et al*.). The red grouping in the dendrogram represents breast tumors with high expression of the signature genes. The Wang *et al*. dataset [26] includes data from 209 patients, and used the same Hu133A-Affymetrix microarray platform used by Frasor *et al*. [10]. All 29 genes from the 14-3-3ζ signature were retrieved and used for data mining. Unsupervised clustering analysis identified the red group (Signature High, *P *= 0.001 Kaplan-Meier, Figure 2b) Panel I as a poor prognosis group driven by high expression of the signature genes.

In a similar fashion, we analyzed the ER-positive breast tumors (*n *= 49) included in the van't Veer dataset [17]. Given the different microarray platform used (Hu25K-Agilent), a reduced number of genes were retrieved, 17 out of the 29 genes in the 14-3-3ζ gene signature. The signature genes not retrieved by our analysis were not present on those arrays. However, the subset of patients characterized by high expression of the 14-3-3ζ signature showed a significantly earlier relapse (Figure 2b, Panel II). We also examined the dataset of Sorlie *et al*. [16,27], which used cDNA Stanford arrays containing 8,102 genes. Expression data for 19 genes of the gene signature were recovered and used for the analysis. (The signature genes not recovered were not present on these arrays.) The findings confirmed once again that overexpression of the 14-3-3ζ signature was significantly associated with a poorer disease-free survival (Figure 2b, Panel III).

### Breast cancer subtypes and the 14-3-3ζ gene signature

We next examined the distribution of the five major breast cancer molecular subtypes in the set of patients that showed high expression of the 14-3-3ζ gene signature and a poor clinical outcome in the different clinical studies by using a centroid-mediated clustering algorithm. All datasets showed enrichment for luminal-B subtypes in tumors with elevated expression of the 14-3-3ζ signature genes, ranging from 53 to 58% of all tumors (Figure 2c). In addition, 7 to 21% of total ER-positive breast cancers showing high expression of the 14-3-3ζ gene signature were represented by the basal breast cancer subtype. For comparison we also classified tumors characterized by low expression of the 14-3-3ζ gene signature, and found that luminal A was the most abundantly represented molecular subtype in the different datasets (data not shown). When correlated with clinical-pathological features, high level expression of the 14-3-3ζ gene signature was significantly associated with tumor grade, with the luminal-B vs. luminal-A breast cancer subtype, and with metastasis (Figure 2d).

### Tamoxifen selectively upregulates the zeta isoform of 14-3-3 proteins in breast cancer cells

**Figure 3:**
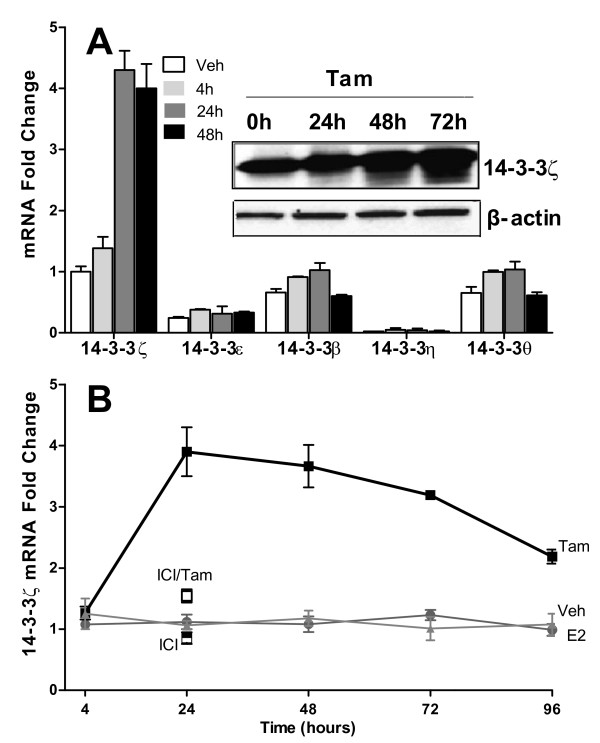
**Regulation of 14-3-3 family members in MCF-7 breast cancer cells upon ligand treatment**. **(a) **Expression of 14-3-3 isoforms at the indicated times (0, 4, 24, 48 hours) after 1 μM tamoxifen (Tam) treatment. 14-3-3σ is expressed at a very low level and is therefore not shown. Inset shows 14-3-3ζ protein evaluated by western blot after Tam treatment for 0 to 72 hours with β-actin as the loading control. **(b) **Cells were cultured in the presence of vehicle (0.1% ethanol), 1 μM Tam, 10 nM E2, 1 μM ICI 182,780 (ICI) alone or ICI in combination with 10 nM Tam for various times. RNAs were measured by real-time PCR..

Based on the findings of a clinical breast cancer gene expression signature associated with high 14-3-3ζ and with risk of recurrence, we undertook studies to examine the effect of perturbing 14-3-3ζ levels on gene regulations and phenotypic properties of ER-positive breast cancer cells. Because 14-3-3ζ belongs to a family of highly conserved proteins, we first examined whether tamoxifen affected regulation of the various members of the 14-3-3 family. Of note, the mRNA level of only the zeta isoform was markedly upregulated by tamoxifen (Figure 3a, *P *= 0.0004), with 14-3-3ζ reaching the maximal mRNA level by 24 hours (Figure 3b) and maximal protein level at 48 to 72 hours after tamoxifen (Figure 3a). Of the other 14-3-3 isoforms, only 14-3-3β showed low but significant (*P *= 0.036) upregulation by tamoxifen. Cotreatment with tamoxifen and the ER antagonist ligand and ER downregulator, ICI 182,780 (ICI), reversed the stimulatory effect of tamoxifen (Figure 3b), indicating the requirement for ER in the upregulation of 14-3-3ζ.

### Functional characterization of the effect of 14-3-3ζ knockdown on the phenotypic properties of ER-positive breast cancer cells

To probe the functional roles of 14-3-3ζ in breast cancer aggressiveness and in antiestrogen resistance, we examined the effect of long-term reduction of 14-3-3ζ on cell phenotypic properties by stable expression of interfering short hairpin shRNA in ER-positive MCF7 cells. We subcloned the human U6 promoter [28] into the plasmid vector pRNAtin and five shRNAs targeting the 3'-noncoding region of 14-3-3ζ and a non-targeting control shRNA were designed. Several clones showed 14-3-3ζ reduction, but only two showed a good level of reduction of 14-3-3ζ (reduction by approximately 60% or 70%). We assume this likely reflects our findings, presented in more detail below, that depletion of 14-3-3ζ greatly slows cell growth and induces apoptosis. Hence, cells are unable to survive in the complete absence of this protein.

**Figure 4:**
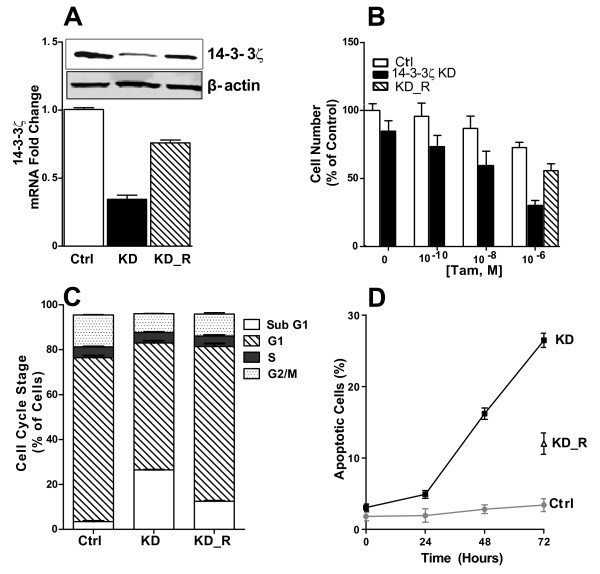
**Characterization of the phenotypic properties of MCF-7 cells with knockdown of 14-3-3ζ**. **(a) **14-3-3ζ RNA and protein levels were evaluated by quantitative PCR and western blot in cells stably expressing control shRNA (Ctrl) or 14-3-3ζ shRNA knockdown (KD) and in KD cells transfected with wild type 14-3-3ζ (KD_R, knockdown and reexpression). **(b) **Cell viability in response to different concentrations of tamoxifen (Tam) for 48 hours for Ctrl or 14-3-3ζ KD or KD_R cells. Cell number for vehicle-treated control cells is set as 100%. **(c) **Percentage of cells in the different cell cycle stages for Ctrl and 14-3-3ζ KD or KD_R cells treated with 1 μM Tam for 72 hours. **(d) **Percentage of apoptotic cells in Ctrl, 14-3-3ζ KD, and KD_R cells treated with 1 μM Tam.

We undertook characterization of the two clones showing a downregulation by about 60 to 70%, and found similar trends, so we present data only for the clone showing the greatest 14-3-3ζ depletion (Figure 4a). These cells, referred to as 14-3-3ζ KD, showed 35% and 30% of the parental cell content of 14-3-3ζ at the RNA and protein level, respectively (Figure 4a). This knockdown of 14-3-3ζ did not affect the levels of other 14-3-3 isoforms (data not shown). To validate the specificity of our shRNA knockdown, which was targeted to the 3'-UTR of 14-3-3ζ, we re-expressed 14-3-3ζ cDNA that did not contain the 3'-UTR (denoted KD_R, knockdown and re-expression). Re-expression of 14-3-3ζ in the KD cells substantially restored 14-3-3ζ mRNA and protein (KD_R, Figure 4a).

Cells with downregulation of 14-3-3ζ showed enhanced sensitivity to tamoxifen inhibition of cell viability (Figure 4b). Decreased proliferation of the 14-3-3ζ KD cells was explained by a marked increase of cells in the sub-G1 phase of the cell cycle and a decrease of cells in G1 and G2/M phases (Figure 4c), based on flow cytometric analysis. Moreover, apoptosis was found to be greatly increased with time of tamoxifen treatment in 14-3-3ζ depleted cells compared with control cells (Figure 4d).

### 14-3-3ζ knockdown impacts FOXM1 and 14-3-3ζ signature genes

**Figure 5:**
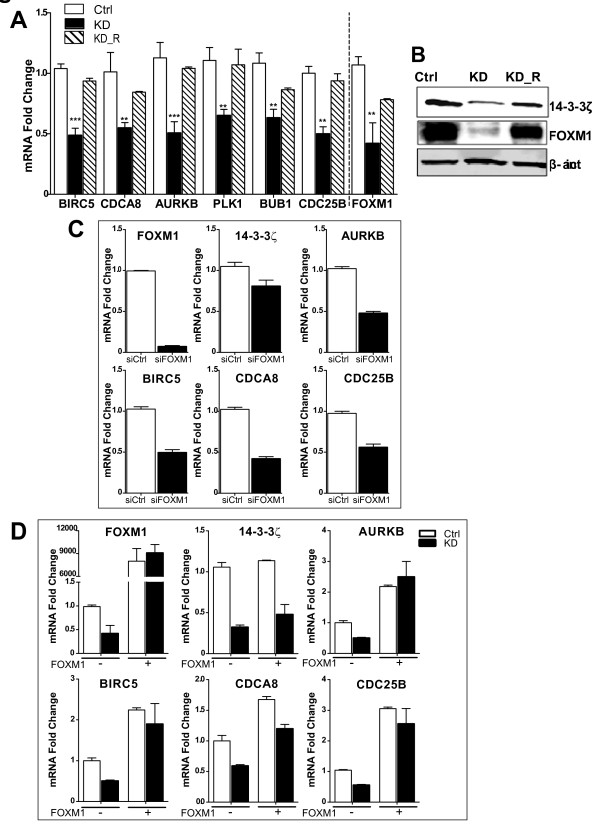
**Expression of 14-3-3ζ and associated signature genes in 14-3-3ζ knockdown MCF-7 cells**. **(a) **Real-time PCR for six signature genes in control (Ctrl), 14-3-3ζ knockdown (KD) and KD cells with reexpression of 14-3-3ζ (KD_R). Reduced levels of 14-3-3ζ correlated with decreased levels of all investigated genes (black bars; *** *P *< 0.001, ** *P *< 0.01). 14-3-3ζ KD cells with re- expression of 14-3-3ζ (hatched bars) showed gene expression similar to the control (Ctrl) cells. 14-3-3ζ KD was also associated with decreased FOXM1 mRNA that was reversed with reexpression of 14-3-3ζ. **(b) **14-3-3ζ and FOXM1 protein in Ctrl, 14-3-3ζ KD, and with 14-3-3ζ KD and reexpression. Western blots are shown with actin as loading control. **(c) **Impact of siFOXM1 treatment (black bars) on FOXM1 and expression of 14-3-3ζ and 14-3-3ζ signature genes. **(d) **Re-expression of FOXM1(+FOXM1) in Ctrl and 14-3-3ζ KD cells and its effect on 14-3-3ζ and signature genes. Minus indicates no added FOXM1.

Next, we selected several genes from the 14-3-3ζ signature and monitored their levels in cells with stable 14-3-3ζ knockdown. Of note, reduction of 14-3-3ζ was associated with a significant reduction in the expression of signature genes, including BIRC5/Survivin, CDCA8, AURKB, PLK1, BUB1, and CDC25B, and this was reversed by restoration of 14-3-3ζ (Figure 5a). Further, we inspected the cellular level of FOXM1, a transcription factor known to regulate expression of cell cycle genes [29,30], including some of our signature genes. In 14-3-3ζ KD cells, we observed a significant decrease in FOXM1 mRNA and a particularly marked reduction of FOXM1 protein correlating with low levels of 14-3-3ζ (Figure 5a and 5b). Further, re-expression of 14-3-3ζ (KD_R cells) substantially restored the level of FOXM1 (Figure 5a and 5b).

By transient knockdown of FOXM1 with siRNA (to 10% of control level, Figure 5c), we observed a marked reduction of AURKB, BIRC5, CDCA8, and CDC25B but little impact on 14-3-3ζ (Figure 5c), indicating that the major regulatory effect of FOXM1 on these genes is downstream of 14-3-3ζ. To explore this further, we treated cells with FOXM1-expressing adenovirus and found that elevation of FOXM1 had no effect on 14-3-3ζ levels in either control or 14-3-3ζ KD cells (Figure 5d), whereas the overexpression of FOXM1 increased expression of the four signature genes and fully abrogated the effect of 14-3-3ζ knockdown (Figure 5d). This pattern of regulation provides support for the regulatory effect of FOXM1 on these genes being downstream of 14-3-3ζ.

### Downregulation of 14-3-3ζ in tamoxifen-resistant cells restores sensitivity to the inhibitory effects of antiestrogens

**Figure 6:**
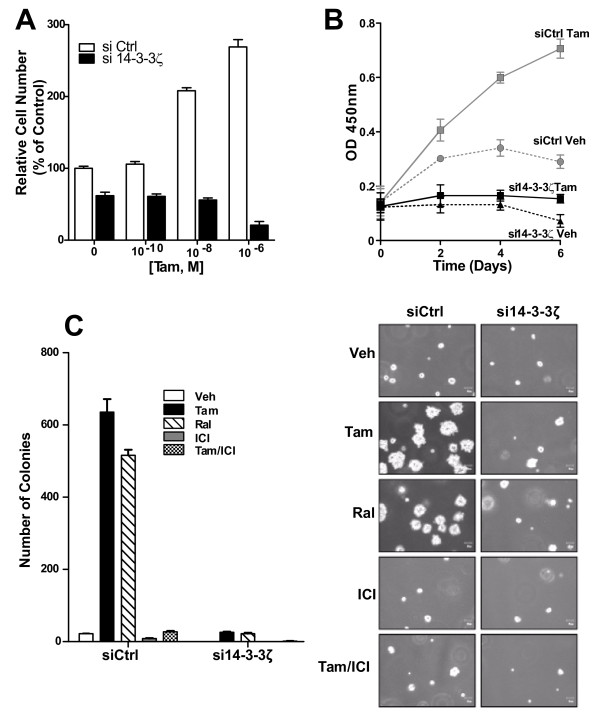
**Effects of 14-3-3ζ on viability and colony formation of tamoxifen-resistant (Tam**^**R**^**) MCF-7 cells**. **(a) **Sensitivity to tamoxifen measured as a function of cell viability in response to increasing concentrations of ligand for 96 hours. Vehicle-treated control cells were set at 100%. **(b) **Cell viability monitored over time in Tam^R ^cells with siCtrl or si14-3-3ζ and treatment with tamoxifen (Tam) for the days indicated. **(c) **Colony formation of Tam^R ^Ctrl siRNA or 14-3-3ζ siRNA cells in soft agar after 15 days in the presence of vehicle, Tam (1 μM), raloxifene (1 μM), fulvestrant (ICI 182,780) (1 μM) and Tam plus ICI (10 nM and 1 μM). Colonies were stained, counted, and photographed.

To assess the role of 14-3-3ζ in antiestrogen resistance, we used a tamoxifen-resistant breast cancer cell line (Tam^R ^cells) generated in our laboratory [21]. 14-3-3ζ was three times higher in these resistant cells than in the parental MCF7 cells (data not shown), and tamoxifen elicited growth stimulation, rather than growth inhibition, in these cells (Figures 6a and 6b). Knockdown of 14-3-3ζ eliminated tamoxifen stimulation of proliferation and also reduced control cell proliferation (Figure 6b). 14-3-3ζ knockdown also greatly reduced anchorage-independent growth of antiestrogen-resistant cells which grew well in the presence of tamoxifen and raloxifene without 14-3-3ζ knockdown (Figure 6c).

**Figure 7:**
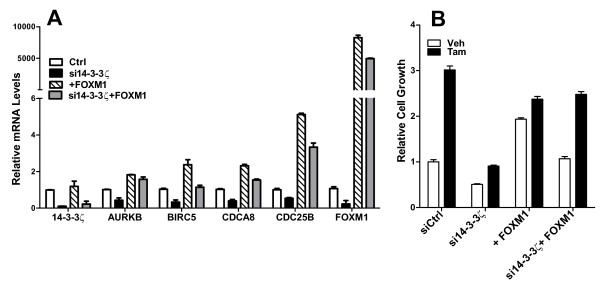
**Impact of 14-3-3ζ knockdown and FOXM1 expression on signature genes and proliferation of tamoxifen-resistant cells**. **(a) **Expression of 14-3-3ζ, 14-3-3ζ signature genes, and FOXM1 in MCF-7 tamoxifen-resistant cells with siRNA knockdown of 14-3-3ζ and/or overexpression of FOXM1. **(b) **Cell viability monitored after 96 hours of vehicle or tamoxifen (Tam) treatment in tamoxifen-resistant cells with knockdown of 14-3-3ζ and/or overexpression of FOXM1. Control cells transfected with control siRNA were taken as 100%.

With knockdown of 14-3-3ζ in tamoxifen-resistant cells, we observed a downregulation of the 14-3-3ζ signature genes and a marked reduction in FOXM1 (Figure 7a, black bars), and also a suppression of control (veh) cell proliferation and a greatly reduced stimulation of proliferation by tamoxifen (Figure 7b). With FOXM1 overexpression (Figure 7a, hatched bars), expression of 14-3-3ζ signature genes was increased (Figure 7a), and this FOXM1 elevation resulted in an increase in control cell proliferation with only a limited further stimulation by tamoxifen (Figure 7b). When 14-3-3ζ was depleted from cells and FOXM1 was overexpressed, expression of the signature genes was restored to or even increased above the control level (Figure 7a, grey bars), and basal proliferation and stimulation of proliferation by tamoxifen were restored (Figure 7b).

### Effect of 14-3-3ζ overexpression or knockdown on markers of hormone resistance

**Figure 8:**
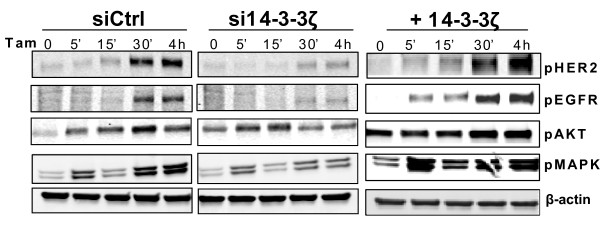
**14-3-3ζ status impacts the activation of key signaling proteins**. Phosphorylation of human epidermal growth factor receptor 2 (HER2), epidermal growth factor receptor (EGFR), AKT/Protein kinase B (AKT/PKB), and mitogen activated protein kinase (MAPK) was investigated by western blot in tamoxifen-resistant cells following siRNA knockdown or overexpression of 14-3-3ζ and treatment with 1 μM tamoxifen (Tam).

As it is known that enhanced activation of growth factor receptors and downstream kinases can underlie tamoxifen resistance, we examined the impact of 14-3-3ζ status on possible changes in these signaling proteins. We modulated the levels of 14-3-3ζ by adenovirus overexpression or knockdown by RNA interference in tamoxifen-resistant cells, and we monitored over time the status of phosphorylated HER2, EGFR, and downstream signaling kinases AKT and MAPK in cells treated with tamoxifen. Of note, with elevated levels of 14-3-3ζ, the tamoxifen-resistant cells showed enhanced phosphorylation of HER2, EGFR, and MAPK, with lesser impact on pAKT (Figure 8, right). The opposite effects were observed when cells were depleted of 14-3-3ζ, namely suppression of activation of HER2, EGFR, AKT, and MAPK (Figure 8, middle). Hence, 14-3-3ζ plays an important role in modulating the activation status of these key receptors and protein kinases.

## Discussion

Endocrine therapies initially provide benefit in many of the approximately 70% of breast cancers that are ER-positive, but the effectiveness of endocrine therapies is often lost with time because resistance to treatment develops. In this study, we show that 14-3-3ζ is a critical factor promoting endocrine resistance. It is upregulated in endocrine-resistant breast cancer and its depletion reverses resistance and restores sensitivity to endocrine treatments.

In probing the functional dimensions of the roles 14-3-3ζ plays in endocrine resistance, we have identified a gene signature associated with high expression of 14-3-3ζ, based on microarray datasets from approximately 400 women with ER-positive breast tumors, and we find that this gene signature is correlated with higher tumor grade, increased metastasis, and risk of early recurrence. Up or downregulating the level of 14-3-3ζ greatly impacted the phenotypic properties of breast cancer cells, including their proliferation, apoptosis, and endocrine sensitivity. Notably, downregulation of 14-3-3ζ restored sensitivity to endocrine treatments in endocrine-resistant breast cancer cells and reduced the expression of signature genes associated with proliferation and survival, effects that were reversed by re-expression of 14-3-3ζ. Thus, 14-3-3ζ appears to function as a key therapeutic target whose downregulation could improve response to endocrine therapies.

### A gene signature and breast cancer molecular subtypes associated with 14-3-3ζ overexpression and poor patient outcome

Using a training set of 67 adjuvant tamoxifen-treated ER-positive breast tumors, we identified, by a supervised analysis, a set of 29 genes that strongly correlated with expression of 14-3-3ζ. By taking advantage of several publicly available large independent breast cancer datasets, we confirmed the ability of the 14-3-3ζ signature to predict clinical outcome. These four large datasets represent a combined total of nearly 400 breast cancer patients with ER-positive tumors. The gene signature was robustly represented by cell cycle-related genes, and factors such as BIRC5/survivin, that have been shown to play important roles in mitosis and to promote cell survival [26,29,31,32].

Our studies are the first to reveal the detrimental role of 14-3-3ζ in endocrine therapy resistance, and they provide a molecular basis for our observation of a poor clinical outcome for women with breast cancers having high expression of 14-3-3ζ. Our findings reveal that 14-3-3ζ is associated with a gene signature rich in genes that encode proteins with central roles in mitosis and the segregation of chromosomes during cell division. The enzyme aurora B kinase, which is part of the chromosome passenger complex, and the protein kinase BUB1 have through recent studies been documented as essential for accurate chromosome inheritance at mitosis. Aurora B kinase appears to act as a fidelity checkpoint factor for mitosis by reversibly phosphorylating target proteins at the centromere and kinetochore [33-35]. BUB1 phosphorylation of a specific threonine in histone H2A has been implicated in the recruitment of the chromosome passenger complex to centromeres [35]. Survivin, also part of our gene signature, binds to aurora B in the chromosome passenger complex, contributing to events that control the normal segregation of chromosomes during cell division. Alterations in the production of these factors, which we observed as a consequence of upregulation of 14-3-3ζ by tamoxifen and associated with the development of endocrine resistance, might thereby also impair proper chromosome segregation and affect cell viability and tumor progression. Indeed, we found that changes in the levels of 14-3-3ζ by knockdown or overexpression had marked effects on cell viability, apoptosis, and on the cell cycle in MCF-7 tamoxifen-resistant cells, and also in ER-positive and HER2-positive BT474 cells based on preliminary studies.

Our studies also uncovered a previously unknown relation between 14-3-3ζ and FOXM1, with 14-3-3ζ playing a crucial role in regulating FOXM1. This was observed in MCF-7 parental and tamoxifen-resistant cells, as shown in this study, and also in ER-positive HER2-positive BT474 breast cancer cells (data not shown). Thus, knockdown of 14-3-3ζ brought about an almost complete loss of cellular FOXM1 protein, which was restored upon re-expression of 14-3-3ζ. Further, the regulation of 14-3-3ζ mitosis-signature genes appears to result from 14-3-3ζ control of FOXM1, because increasing FOXM1 levels in the context of 14-3-3ζ knockdown effected a parallel restoration of the expression of these genes. Thus, 14-3-3ζ appears to function upstream of FOXM1 in regulating these signature genes. Some of the effects of 14-3-3ζ status on the cell cycle might reflect changes in the cellular level of FoxM1, which is known to regulate genes involved in G2/M, some of which were investigated in this study including BIRC5, AURKB, CDCA8, CDC25B, and PLK1 [29,30].

The majority of ER-positive breast tumors overexpressing 14-3-3ζ were of the luminal B subtype, tumors with a poorer outcome compared with luminal A. Consistent with this, comparative genomic hybridization analyses have indicated that one of the most recurrent alterations in luminal B tumors is gain/amplification of the 8q region that harbors 14-3-3ζ (8q22) [36]. In addition to the prominent association of 14-3-3ζ with ER-positive luminal B tumors, ca. 12% were basal breast cancers, another subtype with a poor prognosis. Collectively, our observations in primary breast tumors and in breast cancer cells *in vitro *provide evidence that the overexpression of 14-3-3ζ and the associated 14-3-3ζ gene signature identify a subgroup of ER-positive tumors most likely to be resistant to endocrine therapies and to show early recurrence. In addition, our studies reveal that 14-3-3ζ expression can also be increased as a consequence of tamoxifen treatment, and therefore, ironically, that tamoxifen itself, through upregulation of 14-3-3ζ, may be contributing to the development of endocrine resistance.

### Broad impact of 14-3-3ζ on key cellular activities and signaling pathways

14-3-3ζ status had a great impact on cell signaling pathways and the molecular properties of breast cancer cells. With high 14-3-3ζ, cells showed enhanced activation of EGFR, HER2, MAPK, and AKT, and increased anchorage-dependent and independent growth. These activities were suppressed by downregulation of 14-3-3ζ. Thus, 14-3-3ζ increases signaling through a variety of growth factor receptors and protein kinase pathways, stimulating a more robust and temporally prolonged activation of these pathways to promote survival and anti-apoptotic signaling, and enhance the endocrine resistance of breast cancer cells.

14-3-3ζ is a member of a highly conserved family of 14-3-3 proteins, and it functions as a scaffold or platform that regulates the activity and stability of interacting proteins by binding to their phosphoserine and phosphothreonine motifs. It is noteworthy that 14-3-3ζ is the major form expressed in breast tumors and in ER-positive breast cancer cells, and it was the only 14-3-3 isoform to show high upregulation by tamoxifen. The broad effects of 14-3-3ζ might indeed be expected for a scaffold/adaptor protein that serves as a critical convergence factor in these signaling pathways, having known interactions with EGFR [13], HER2 [13], and PKC [11], as well as additional signaling components such as RAF-1 and β-catenin [12,14]. Our observations now add regulation of FOXM1 as another important aspect of 14-3-3ζ activity in breast cancer and endocrine resistance.

### 14-3-3ζ as a key marker of endocrine resistance and a therapeutic target for endocrine therapy sensitization

As 14-3-3ζ is overexpressed in breast cancers with a poor prognosis, and its elevated expression is associated with activation of growth factor and mitogenic signaling pathways and with endocrine resistance, our data imply that 14-3-3ζ should serve as a marker of resistance and a key therapeutic target for endocrine therapy sensitization and effective tumor suppression. Resistance to endocrine therapies is associated with enhanced signaling through growth factor receptor and downstream kinase pathways including MAPK and AKT [6,7,9,37-39]. Further, these signaling cascades result in the activation of additional kinases such as polo-like kinase 1 and the cyclin-CDKs, which are part of the 14-3-3ζ gene signature.

## Conclusions

In summary, we find 14-3-3ζ to be a key marker for risk of failure on endocrine therapy and show that its elevated expression promoted resistance to endocrine therapies, whereas its downregulation slowed proliferation, enhanced apoptosis, and increased the sensitivity of breast cancer cells to endocrine treatment. From our studies and those of others [10,15,40-43], 14-3-3ζ is emerging as a critical factor that has major impact on multiple forms of cancer therapy, endocrine therapies, and certain chemotherapies as well [44]. Our findings provide new mechanistic insights through definition of a gene signature and molecular phenotype associated with overexpression of 14-3-3ζ that contributes to endocrine resistance. Targeting 14-3-3ζ and the factors it regulates, such as FOXM1, should prove beneficial in delaying the development of endocrine resistance and in reversing resistance, and should allow more effective treatment of patients whose tumors overexpress 14-3-3ζ and are at high risk for disease recurrence.

## Abbreviations

ER: estrogen receptor; FDR: false discovery rate; KD: knockdown; PCR: polymerase chain reaction; PI: propidium iodide; SAM: statistical analysis of microarrays; SERD: selective estrogen receptor downregulator; SERM: selective estrogen receptor modulator.

## Competing interests

The authors declare that they have no competing interests.

## Authors' contributions

AB conceived and designed the studies, carried out the experiments and data analysis, interpreted the data and wrote drafts of the manuscript. BC carried out some of the experimental studies and data analysis. BSK conceived and designed the studies, analyzed and interpreted the data, and wrote the manuscript. All authors read, made suggestions, and approved the final manuscript.
